# Pharmacokinetics of epinephrine in patients with septic shock: modelization and interaction with endogenous neurohormonal status

**DOI:** 10.1186/cc7972

**Published:** 2009-07-21

**Authors:** Imad Abboud, Nicolas Lerolle, Saik Urien, Jean-Marc Tadié, Françoise Leviel, Jean-Yves Fagon, Christophe Faisy

**Affiliations:** 1 Medical Intensive Care Unit, Hôpital Européen Georges Pompidou, Assistance Publique-Hôpitaux de Paris, Université Paris – Descartes, Paris, France; 2 E.A. 3620, CIC-0109 Cochin-Necker Paris Descartes, Unité de Recherche Clinique, Tarnier Hospital, Assistance Publique-Hôpitaux de Paris, Université Paris – Descartes, Paris, France; 3 Department of Physiology, Hôpital Européen Georges Pompidou, Assistance Publique-Hôpitaux de Paris, Université Paris – Descartes, Paris, France

## Abstract

**Introduction:**

In septic patients, an unpredictable response to epinephrine may be due to pharmacodynamic factors or to non-linear pharmacokinetics. The purpose of this study was to investigate the pharmacokinetics of epinephrine and its determinants in patients with septic shock.

**Methods:**

Thirty-eight consecutive adult patients with septic shock were prospectively recruited immediately before epinephrine infusion. A baseline blood sample (C_0_) was taken to assess endogenous epinephrine, norepinephrine, renin, aldosterone, and plasma cortisol levels before epinephrine infusion. At a fixed cumulative epinephrine dose adjusted to body weight and under steady-state infusion, a second blood sample (C_1_) was taken to assess epinephrine and norepinephrine concentrations. Data were analyzed using the nonlinear mixed effect modeling software program NONMEM.

**Results:**

Plasma epinephrine concentrations ranged from 4.4 to 540 nmol/L at steady-state infusion (range 0.1 to 7 mg/hr; 0.026 to 1.67 μg/kg/min). A one-compartment model adequately described the data. Only body weight (BW) and New Simplified Acute Physiologic Score (SAPSII) at intensive care unit admission significantly influenced epinephrine clearance: CL (L/hr) = 127 × (BW/70)^0.60 ^× (SAPS II/50)^-0.67^. The corresponding half-life was 3.5 minutes. Endogenous norepinephrine plasma concentration significantly decreased during epinephrine infusion (median (range) 8.8 (1 – 56.7) at C_0 _vs. 4.5 (0.3 – 38.9) nmol/L at C_1_, *P *< 0.001).

**Conclusions:**

Epinephrine pharmacokinetics is linear in septic shock patients, without any saturation at high doses. Basal neurohormonal status does not influence epinephrine pharmacokinetics. Exogenous epinephrine may alter the endogenous norepinephrine metabolism in septic patients.

## Introduction

Symptomatic treatment of septic shock is primarily aimed at improving hemodynamic and oxygen transport variables in order to restore organ perfusion. Hemodynamic stabilization in septic shock is achieved through adequate volume resuscitation and the use of vasoactive agents. In the past decade, dopamine and norepinephrine were considered to be the drugs of choice to increase arterial pressure, rather than epinephrine, which alters metabolism substantially [1-4]. However, Annane and colleagues reported similar efficacy and safety when comparing norepinephrine with epinephrine combined with dobutamine [5].

Clinical experience suggests a considerable intra and inter-patient variability of arterial pressure in response to epinephrine infusion. A ceiling effect frequently occurs in more severe cases, requiring maximum epinephrine doses in which further increases in infusion rate lead to modest or no increase in blood pressure. This unpredictable response may be due to pharmacodynamic factors or non-linear pharmacokinetics. Free radicals and nitric oxide produced in sepsis are able to oxidize and neutralize catecholamines and may therefore enhance catecholamine clearance [6,7]. In a rat model of sepsis, inhibition of free radical production prevented the drop in catecholamine blood concentrations and hypotension [7]. In addition, it was suggested that proinflammatory mediators may also neutralize catecholamines [7]. Conversely, liver and kidney dysfunction may lower epinephrine clearance, and exogenously administered epinephrine may accelerate the release of endogenous epinephrine via the sympathetic nerve endings and the adrenal medulla [8,9]. Finally, adrenal status and physiological doses of hydrocortisone influence the pressor response to vasoactive drugs [10]. The mechanisms by which glucocorticoid hormones modulate the vascular response to vasopressors are not well known, and a pharmacokinetic alteration may occur.

The purpose of this study was to investigate the pharmacokinetics of epinephrine in patients with septic shock, assuming that a ceiling may be observed at high doses. In addition, we assessed whether endogenous neurohormonal status alters epinephrine pharmacokinetics. We demonstrated that epinephrine pharmacokinetics is linear in septic shock patients, without any saturation at high doses, and that higher disease severity is associated with lower epinephrine clearance. Furthermore, basal neurohormonal status did not influence epinephrine pharmacokinetics.

## Materials and methods

This prospective study was conducted in the 18-bed medical intensive care unit (ICU) of a tertiary teaching hospital in France from January to June 2006. The Ethics Committee of the Société de Réanimation de Langue Française approved the study and waived the need for written informed consent. Participants, or immediate family members if a patient was unable to respond, were informed of the objectives of the procedure and oral consent was obtained. All consecutive adult patients with septic shock were eligible. Septic shock was defined by the presence of infection, dysfunction in at least one organ and fluid refractory hypotension (mean arterial pressure below 65 mmHg) requiring the administration of vasopressor agents [11]. Exclusion criteria were pregnancy, renal replacement therapy during the study period and administration of catecholamines in the 24 hours preceding enrolment. Patients were included in the study when the attending physician considered vasopressor infusion. They were thus enrolled before the onset of infusion.

### Intervention

As standardized in our ICU, epinephrine was used as the first-line vasopressor. Epinephrine (diluted to 1:10 in 0.9% saline) was started intravenously using a programmable syringe pump (Pilote IEC, Fresenius-Vial, Bressins, France) at a rate of 0.15 μg/kg/min. The infusion rate was then adjusted to obtain a mean arterial pressure between 65 and 75 mmHg. Of note, continuous epinephrine infusion is required in septic shock patients to maintain arterial pressure until the patient's hemodynamic status improves, generally over several hours or days; thus duration of perfusion or total cumulative dose cannot be predicted. In this study, neither intravenous hydrocortisone nor recombinant human activated protein C were used, and epinephrine was the exclusive catecholamine used.

### Blood sampling

An initial blood sample (C_0_) was drawn within the 15 minutes preceding epinephrine infusion. A second blood sample (C_1_) was drawn when the cumulative epinephrine dose adjusted to body weight (BW) reached a threshold fixed arbitrarily at 0.15 mg/kg, provided the epinephrine infusion rate remained steady and fluid loading was not used in the preceding 15 minutes. In the case of modification of the epinephrine infusion rate or fluid loading in the 15 minutes preceding the threshold dose, C_1 _was delayed until a 15-minute period of stability for the infusion rate was obtained. The 15-minute steady-state interval was chosen according to epinephrine plasmatic half-life in healthy subjects [12]. The epinephrine infusion rate (mg/hr) at C_1 _time was recorded. Blood drawn on C_0 _was used to assess plasma levels of endogenous adrenal axis hormones: epinephrine, norepinephrine, renin, aldosterone, and cortisol. Blood drawn on C_1 _allowed for the measurement of epinephrine and norepinephrine plasma levels.

### Sample handling

Blood assigned to catecholamine assays was sampled in EDTA-tubes and immediately centrifuged at 3000 g for five minutes. The plasma samples were then immediately stored at -80°C before assay. Blood assigned to renin and aldosterone assays was also sampled in EDTA-tubes and centrifuged at 3000 g for five minutes. The plasma was then separated and stored at -20°C. Blood assigned for cortisol assays was allowed to clot at room temperature for 30 minutes, then centrifuged at 3000 g for five minutes. Samples were stored at -20°C.

### Assays

Epinephrine and norepinephrine concentrations were measured in plasma using high-pressure liquid chromatography (HPLC) with coulometric detection [13,14]. The limit of quantification (defined by a variability between measurements of <10%) for HPLC was 0.10 nmol/L. The epinephrine concentration measured on C_1 _was the sum of endogenous and exogenous epinephrine, as the two compounds are strictly identical with regard to chromatographic detection. Plasma aldosterone was measured in duplicate by RIA using a commercial kit from the Diagnostic Products Corporation (Los Angeles, CA, USA). Renin and cortisol concentrations were measured on a fully automated chemiluminescence analyzer (LIAISON^® ^Analyzer, DiaSorin S.p.A, Salluggia-Vercelli, Italy). Plasma renin concentration was measured with a Direct Renin assay. This two-site immunometric assay was calibrated according to World Health Organization reference material (National Institute for Biological Standards and Control, code 58/356). Normal ranges in the supine position for plasma renin, aldosterone, and cortisol concentrations were 10 to 25 mU/L, 80 to 400 pmol/L, and 330 to 500 nmol/L, respectively.

### Patient data

Baseline demographic data included sex, age, BW, new simplified acute physiology score (SAPS) II at study inclusion [15], ICU length of stay before inclusion and cause of septic shock. The volume of fluid administered for resuscitation of shock before C_1 _was recorded. Invasive blood pressure and heart rate were collected at C_0 _and C_1_.

### Population pharmacokinetics modeling of epinephrine

Data were analyzed using the nonlinear mixed effect modeling software program NONMEM version VI driven by Wings for Nonmem (WfN, Free Software Foundation, Boston, MA, USA) [16]. The FOCE method was used. This method allows for the estimation of both the pharmacokinetic and statistic parameters of the model, that is, the elimination clearance is estimated along with its corresponding between-subject variability (BSV). Any residual variability, including measurement errors, can also be estimated. Epinephrine pharmacokinetics was ascribed to a one-compartment open model with first order elimination. Parameters for the model were plasma clearance (CL), volume of distribution (V), and baseline rate of epinephrine infusion (R0). The R0 parameter allowed us to take into account the baseline epinephrine concentration at C_0_. BSVs were assumed to be exponential and their variances and standard deviations (SD) were denoted as ω^2^CL and ωCL, respectively. Covariances were also estimated. When a full covariance matrix could not be estimated, the following algorithm was applied: ω^2^CL was always retained; and if the correlation between terms was low, it was fixed at 0.

Proportional, additive or mixed error models were investigated to describe any residual variability. The main covariates of interest in the population were sex, age, BW, SAPS II, volume of liquid infused, and plasma hormone concentrations. Parameter estimates were standardized for a mean standard covariate using an allometric model: P_i _= P_STD _× (BW_i_/BW_STD_)^θBW ^where P_STD _and θ_BW _are the standard parameter value for a patient with the standard BW value and the power parameter estimate for BW, and P_i _and BW_i _are the parameter and BW of the ith individual. Graphical evaluation of goodness-of-fit was mainly assessed by observed vs. predicted concentrations and weighted residuals vs. time and/or weighted residuals vs. predicted concentrations. The final population model was also ascertained by normalized prediction distribution error metrics [17]. The stability of the model and accuracy of the parameters were assessed by a bootstrap method implemented in Wings for Nonmem [18] and diagnostic graphics and distribution statistics were obtained using R for Nonmem [18] via the R program [19].

### Statistics

The sample size was calculated based on data from a previous study where the lower limit of the 95% confidence interval of the correlation coefficient *r*^2 ^between epinephrine perfusion rate and its plasma concentration was 0.4. To detect such a correlation (we used a β risk of 20% and an α risk of 5%), 28 patients or more were required. Results are expressed as numbers (%), means ± SD, or medians (range) for data not normally distributed. Analyses were conducted with SPSS 11.5 software (SPSS Inc., Chicago, IL, USA). Wilcoxon and Mann-Whitney tests were applied for comparison of relevant variables. Shapiro-Wilks test was used for the assessment of normality. We considered a difference to be significant when the α risk was < 5% (*P *< 0.05).

## Results

### Patient population

Thirty-eight consecutive patients satisfying the entry criteria were recruited and had their plasma epinephrine concentrations analyzed. We were able to sample C_1 _at the exact threshold dose in all of these patients. Characteristics and demographic data of the enrolled patients are summarized in Table 1. Three C_1 _plasma tubes assigned for epinephrine and norepinephrine blood concentrations were lost due to a technical problem during handling.

**Table 1:** Patientcharacteristics (n = 38)

Characteristic	Value
Age, years, mean ± SD	64 ± 15
Gender, male, n (%)	25 (65.7)
Body weight, kg, mean ± SD	68 ± 19
SAPS II at study inclusion, mean ± SD	64 ± 23
Days in ICU before inclusion, median (range)	1 (1 to 22)
ICU mortality, n (%)	25 (65.7)
Causes of septic shock	
Community-acquired pneumonia	10
Nosocomial pneumonia	12
Mediastinitis	4
Intra-abdominal infection	6
Others	4
Not documented	2

ICU = intensive care unit; SAPS II = new simplified acute physiology score; SD = standard deviation.

### Hemodynamics and plasma hormone concentrations

Epinephrine infusion significantly increased arterial blood pressure and heart rate from C_0 _to C_1_, and was associated with a significant decrease in plasma norepinephrine concentration (Table 2). The median fluid volume administered for shock resuscitation until C_1 _was 4650 ml (range 500 to 11,400). In all patients, 90% of this volume had been administered before C_0_.

**Table 2:** Hemodynamic parameters and plasma hormone concentrations

Parameter	Baseline (C_0_)	0.15 mg/kg Epinephrine (C_1_)	*P *value
Epinephrine infusion rate			
mg/hr	-	2 (0.1 to 7)	
μg/kg/min	-	0.52 (0.026 to 1.7)	
nmol/hr	-	10 090 (545 to 38 208)	
			
Hemodynamics			
Heart rate	109 ± 22	117 ± 22	<0.05
Systolic blood pressure, mmHg	80 ± 13	116 ± 21	<0.0001
Diastolic blood pressure, mmHg	38 ± 9	56 ± 12	<0.0001
Mean blood pressure, mmHg	52 ± 8	76 ± 14	<0.0001
			
Plasma hormone concentrations			
Epinephrine, nmol/L	0.34 (0.10 to 4.3)	95.8 (4.40 to 540)^a^	<0.0001
Norepinephrine, nmol/L	8.8 (0.99 to 56.7)	4.5 (0.30 to 38.9)^a^	<0.0001
Aldosterone, pmol/L	281 (17 to 1478)	-	
Cortisol, nmol/L	762 (170 to 7220)	-	
Renin, UI/L	198 (6.5 to 1246)	-	

Values at baseline (C_0_) and at fixed cumulative dose (0.15 mg/kg) of epinephrine infusion (C_1_). Data are median (range) or mean ± standard deviation.

^a ^Three pieces of data missing.

### Epinephrine pharmacokinetics

**Figure 1:**
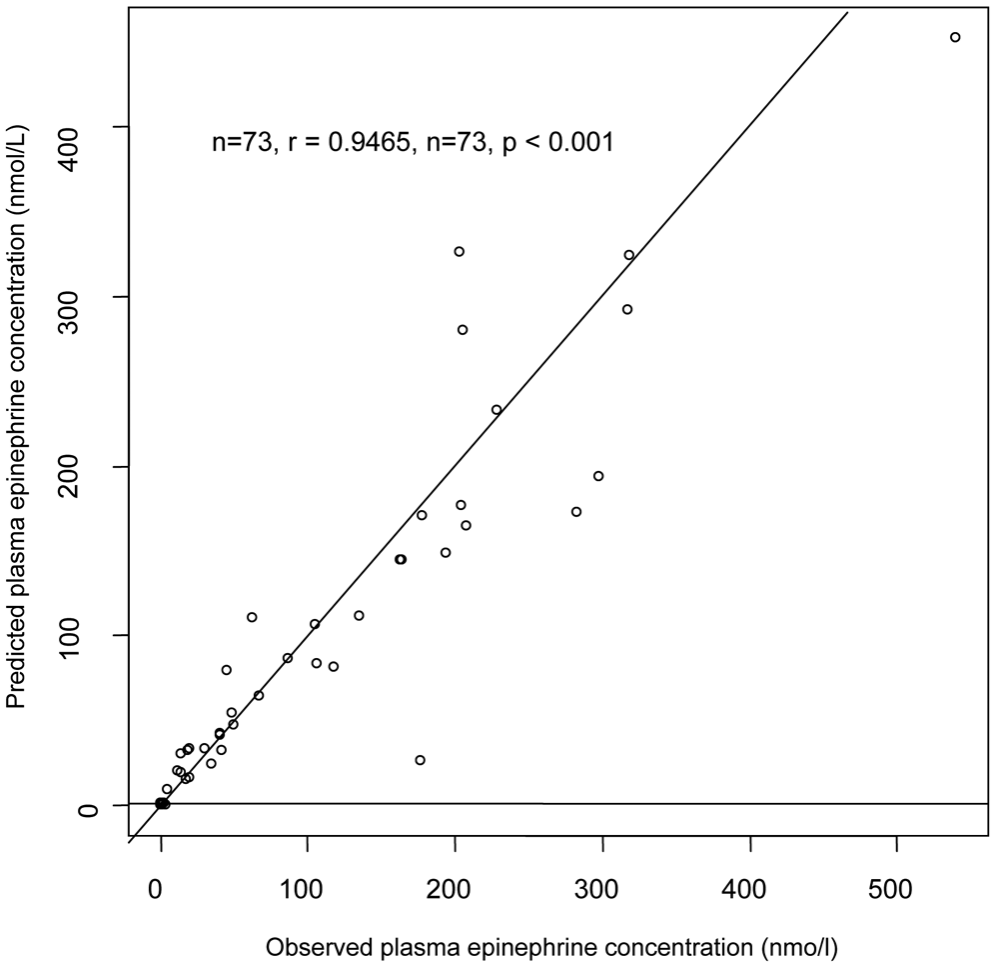
Goodness-of-fit plot for the final model, observed vs. model-predicted epinephrine plateau concentrations. The prediction of the epinephrine plateau concentration at steady state infusion rate is: C_plateau _(nmol/L) = (rate of infusion + R0)/(127 × (BW/70)^0.60 ^× (SAPS II/50)^-0.67^) where R0 (nmol/hr) is the baseline rate of epinephrine infusion rate, BW (kg) is the body weight, and SAPS II is the severity score (new simplified acute physiology score) at intensive care admission.

**Figure 2:**
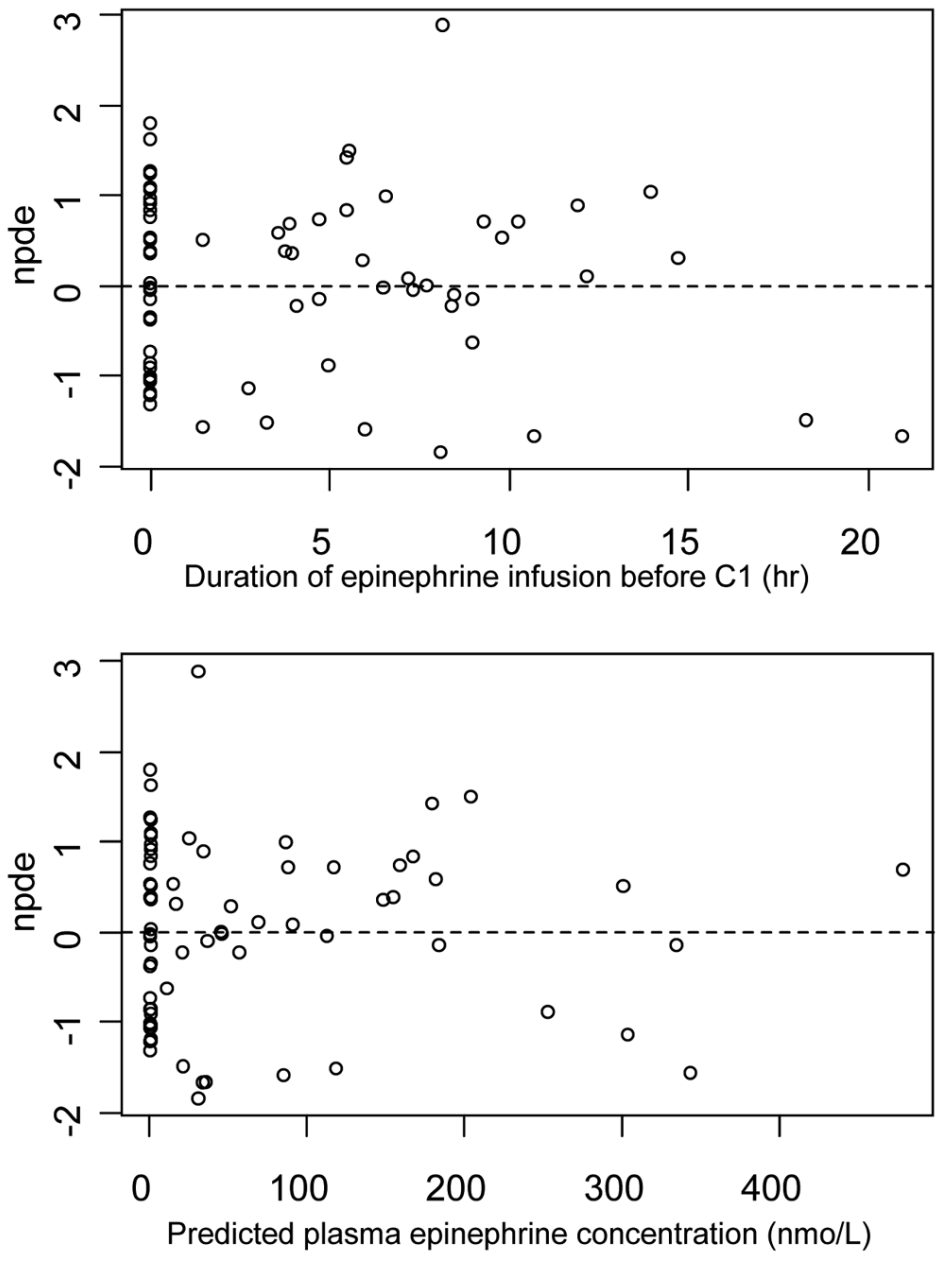
Goodness-of-fit plot for the final model, normalized prediction distribution errors. The upper frame shows normalized prediction distribution errors (npde) vs. duration of epinephrine perfusion (delay C_0 _to C_1_) and the lower frame npde vs. model-predicted concentrations. The npde distribution was not significantly different from normality (*P *= 0.10 by Shapiro-Wilks test). Npde statistics are based on estimates of unbiased means and variances of the observations using 500 Monte Carlo simulations of the final model (the calculations include a de-correlation step of the prediction errors).

Thirty-eight patients and 73 plasma epinephrine concentrations were available for pharmacokinetics evaluation. The median delay from the start of epinephrine infusion to C_1 _was 415 minutes (range 90 to 1260). Despite the fact that the drug dosage was arbitrarily normalized on BW, the infusion rates varied widely at C_1 _due to the significant variations in patients' requirements to achieve hemodynamic goals: there was a greater than 100-fold difference between the lowest and highest rates (Table 2). A one-compartment model adequately described the data. In a first step, only BSV for CL (ωCL) and an additive component for the residual variability could be estimated. The use of a one-compartment model with Michaelis-Menten (saturable) elimination did not improve fit and could not provide reliable Vmax and Km estimates. However, the accuracy of the residual variability parameter was very poor. Thus, in a second step, the residual variability parameters were fixed as follows: 10% and 0.1 nmol/L for the proportional and additive components, according to the assay quantification as stated in Methods. In this manner, the BSV for CL and R0 could be accurately estimated. The parameter estimates of this basic model were CL, 108 L/hr (ωCL = 0.44), V, 9.1 L, and R0, 43.5 nmol/hr (ωR0 = 1.21). The corresponding half-life was 3.5 minutes. The accuracy of estimates varied from 6% for CL to 36% for V. Only BW and SAPS II at ICU admission significantly influenced epinephrine CL, reducing the objective function value by 20 units and ωCL to 0.33. The final relationship for epinephrine CL was thus: CL_i _(L/hr) = 127 × (BW/70)^0.60 ^× (SAPS II/50)^-0.67 ^where 127 L/hr is the typical CL for an individual weighing 70 kg with a SAPS II of 50. This relationship shows that CL increases with BW and decreases with SAPS II. Using CL, the prediction of the epinephrine plateau concentration at the steady-state infusion rate was: C_plateau _(nmol/L) = (rate of infusion + R0)/(127 × (BW/70)^0.60 ^× (SAPS II/50)^-0.67^). Baseline norepinephrine, aldosterone, renin, and cortisol blood concentrations as well as volume of liquid administered for shock resuscitation had no impact on CL. Table 3 summarizes the final population pharmacokinetics estimates including the bootstrap verification. Figure 1 depicts the predicted versus observed concentrations and Figure 2 shows the corresponding normalized prediction distribution error test for this data.

**Table 3:** Population pharmacokinetic parameters of epinephrine in 38 patients with septic shock and bootstrap statistics

Parameter	Mean	SE (%)	Median^*a*^	5^th ^to 95^th ^percentiles^*a*^
CL (L/hr/70 kg BW/50 SAPS II units)	127	6.0	125	115 to 140
V (L)	7.9	36	7.4	1 to 13
R0 (nmol/h)	43.5	24	43	1 to 65
θ_BW _effect on CL	0.60	33	0.59	0.23 to 0.95
θ_SAPS II _effect on CL	-0.67	21	-0.65	-0.91 to -0.43
BSV(CL) (square root of ω^2^_CL_)	0.33	31	0.30	0.20 to 0.39
BSV(R0) (square root of ω^2^_R0_)	1.23	20	1.23	1 to 2.85
Residual variability, proportional component	0.1^*b*^	NA	NA	NA
Residual variability, additive component, nmol/L	0.1^*b*^	NA	NA	NA

^*a*^Statistics from 387 bootstrap runs (13 abnormal termination runs). Non parametric 90% confidence interval based on the 5^th ^to 95^th ^percentiles.

^*b*^Fixed values.

BSV = between subject variability; BW = body weight; CL = epinephrine clearance; NA = not applicable; R0 = baseline rate of epinephrine infusion; SAPS II = new simplified acute physiology score; SE (%) = standard error of estimate in %; V = epinephrine distribution volume.

θ_BW _effect on CL, CL = 127 (BW/70)^0.60^, the individual CL increases or decreases as a function of BW, it is > 127 L/hr if BW is > 70 kg and < 127 L/hr if BW < 70 kg. θ_SAPSII _effect on CL, CL = 127 (SAPSII/50)^-0.67^, the individual CL increases or decreases as a function of SAPS II score, it is > 127 L/hr if SAPS II is < 50 and < 127 L/hr if SAPS II > 50 units.

## Discussion

In this study, we observed that epinephrine pharmacokinetics were linear in septic shock patients. Epinephrine clearance was dependent on BW and disease severity as estimated by the SAPS II. Increased disease severity was associated with lower clearance. Conversely, basal neurohormonal status was not shown to affect epinephrine pharmacokinetics. Finally, we observed that exogenous epinephrine altered norepinephrine metabolism in septic shock patients.

The linear pharmacokinetics of epinephrine and its decreased clearance with increasing severity of disease did not suggest a significant alteration of infused epinephrine by reactive oxygen species or inflammatory cytokines. However, in the absence of any measurement of the production of reactive oxygen species and oxidized catecholamine metabolites, we cannot exclude a small influence of oxidative stress on epinephrine pharmacokinetics. By contrast, liver and kidney alterations may have reduced the extra-neuronal monoamine transporters, which in turn would decrease epinephrine clearance [8,9]. Additionally, renalase, a newly discovered amine oxidase that specifically degrades circulating catecholamines, is secreted by the kidney and has already been shown to be diminished in chronic renal failure [20]. An involvement of this enzyme in acute conditions merits further study.

The linear pharmacokinetics of epinephrine has already been described in studies of very low doses of epinephrine in adult volunteers [21]. In a small pediatric population, Fisher and colleagues reported a weak correlation between epinephrine doses and concentrations [22]. However, in their study, lower epinephrine infusion rates were used and other catecholamines such as dobutamine and dopamine, which are known to modulate epinephrine pharmacokinetics, were infused concomitantly [22-24]. Notwithstanding, we found an epinephrine clearance close to the epinephrine plasma metabolic clearance rate observed in this study. An influence of SAPS II on catecholamines has already been reported with norepinephrine in septic shock and trauma patients [25]. Wilkie and colleagues reported age-dependent changes in plasma catecholamine metabolic clearance rate in humans [26]. This influence of age is in agreement with our model, because age is a component of SAPS II.

In our study, basal endogenous epinephrine concentrations were higher than those of resting healthy adults [21,27-29] but lower than those found in a previous population of patients with septic shock [30]. This is likely to be because of differences in study populations (surgical patients with septicemia, traumatic, or hemorrhagic shock). The decrease in endogenous norepinephrine concentrations during epinephrine infusion has not been described previously. An epinephrine-induced inhibition of norepinephrine release from sympathetic neuronal endings has been demonstrated [31,32]. A direct feedback control of epinephrine concentration on norepinephrine secretion by the adrenal gland has also been described [33]. Finally, this drop in endogenous norepinephrine suggests the absence of accessory metabolic pathways converting exogenous epinephrine to norepinephrine.

The influence of BW and disease severity on epinephrine pharmacokinetics may account for some of the inter-patient variability in response to epinephrine infusion. The lack of impact of endogenous adrenal axis hormones on epinephrine pharmacokinetics suggests that the improvement in hemodynamics with corticoid substitutive dose in septic shock patients is not related to an alteration of catecholamines pharmacokinetics [10]. Indeed, many pharmacodynamic factors influence the response to catecholamine administration in critically ill patients; previous studies have shown that continuous administration of vasoactive drugs may lead to desensitization of vascular smooth muscle responsiveness and that vascular contractility is depressed by proinflammatory mediators, notably through alterations to adrenergic receptor density and affinity, and by disruption of signal transduction across the cell membrane [25,34-36].

A limit to this study is that epinephrine concentration during infusion was available for only one infusion rate in each patient. However, this was balanced by a relatively large number of septic patients studied. Mainly, patients received epinephrine as the first-line catecholamine, with no other catecholamines. Finally, as we included only patients with septic shock, our results cannot be extended to other etiologies of shock.

## Conclusions

These results show linear epinephrine pharmacokinetics and no saturation at high doses in patients with septic shock. Only BW and severity of illness influenced epinephrine pharmacokinetics. No interaction between exogenous epinephrine and endogenous adrenal axis plasma hormones was observed. These results are a prerequisite for further studies on epinephrine pharmacodynamics.

## Key messages

• In septic shock patients, epinephrine pharmacokinetics is linear.

• Higher BW is associated with higher epinephrine clearance, and increased disease severity is associated with lower clearance.

• Endogenous adrenal axis hormones have no impact on epinephrine pharmacokinetics in these patients.

## Abbreviations

BSV: between-subject variability; BW: body weight; C_0_: initial blood sample; C_1_: second blood sample; CL: plasma clearance; HPLC: high-pressure liquid chromatography; ICU: intensive care unit; R0: baseline rate of epinephrine infusion; SAPS II: new simplified acute physiology score; SD: standard deviation; V: volume of distribution; ω^2^CL: variances of BSV; ωCL: standard deviations of BSV.

## Competing interests

The authors declare that they have no competing interests.

## Authors' contributions

IA participated in the design of the study, enrolled the patients, performed blood sampling, and drafted the manuscript. NL participated in the study coordination, interpreted the data, and drafted the manuscript. SU performed modelization, performed statistical analyses, interpreted the data, and drafted the manuscript. JMT participated in the design and coordination of the study. FL performed hormone concentration measurements, and interpreted the data. JYF participated in the design and coordination of the study. CF conceived the study, participated in its design and coordination, and drafted the manuscript. All authors read and approved the final manuscript.
